# The Y831C Mutation of the *POLG* Gene in Dementia

**DOI:** 10.3390/biomedicines11041172

**Published:** 2023-04-13

**Authors:** Eugenia Borgione, Mariangela Lo Giudice, Sandro Santa Paola, Marika Giuliano, Giuseppe Lanza, Mariagiovanna Cantone, Raffaele Ferri, Carmela Scuderi

**Affiliations:** 1 Oasi Research Institute-IRCCS, 94018 Troina, Italy; 2 Department of Surgery and Medical-Surgical Specialties, University of Catania, 95123 Catania, Italy; 3 Neurology Unit, University Hospital Policlinico “G. Rodolico-San Marco”, 95123 Catania, Italy

**Keywords:** neurodegeneration, movement disorders, dementia, POLG, Y831C

## Abstract

Background: The POLG gene encodes the catalytic subunit of DNA polymerase γ, which is crucial for mitochondrial DNA (mtDNA) repair and replication. Gene mutation alters the stability of mtDNA and is associated with several clinical presentations, such as dysarthria and ophthalmoplegia (SANDO), progressive external ophthalmoplegia (PEO), spinocerebellar ataxia and epilepsy (SCAE), Alpers syndrome, and sensory ataxic neuropathy. Recent evidence has also indicated that POLG mutations may be involved in some neurodegenerative disorders, although systematic screening is currently lacking. Methods: To investigate the frequency of POLG gene mutations in neurodegenerative disorders, we screened a group of 33 patients affected by neurodegenerative diseases, including Parkinson’s disease, some atypical parkinsonisms, and dementia of different types. Results: Mutational analysis revealed the presence of the heterozygous Y831C mutation in two patients, one with frontotemporal dementia and one with Lewy body dementia. The allele frequency of this mutation reported by the 1000 Genomes Project in the healthy population is 0.22%, while in our group of patients, it was 3.03%, thus showing a statistically significant difference between the two groups. Conclusions: Our results may expand the genotype-phenotype spectrum associated with mutations in the *POLG* gene and strengthen the hypothesis of a pathogenic role of the Y831C mutation in neurodegeneration.

## 1. Introduction

Neurodegenerative diseases refer to a wide range of progressive nervous system disorders characterized by the selective loss of neuronal subtypes and specific networks. Generally, neurodegeneration begins in the subcortical regions and spreads to some cortical areas as the disease progresses [1]. Clinically, this results in abnormal execution and control of voluntary movement and in an impairment of memory and other cognitive abilities, eventually leading to movement disorders, dementia, and loss of personal independence. Common examples of neurodegenerative disorders are Alzheimer’s disease (AD), Parkinson’s disease (PD), amyotrophic lateral sclerosis (ALS), Huntington’s disease (HD), frontotemporal dementia (FTD), dementia with Lewy bodies (DLB), and spinocerebellar ataxias [2]. Although these conditions differ in pathophysiology and clinical features, with some mainly causing cognitive impairment and others affecting voluntary movements, they are all disabling and currently without any disease-modifying drugs [3,4,5,6], albeit some non-pharmacological interventions seem to be encouraging [7,8,9,10]. Typically, neuronal loss varies according to the disease, e.g., the hippocampus and other cortical regions in AD, the nigrostriatal regions in PD, the motor neurons in ALS, and the striatal and other cortical regions in HD [11,12,13].

In recent years, increasing findings have highlighted the role of impaired mitochondrial functioning in different neurodegenerative diseases, including AD, PD, ALS, and HD [14,15,16]. Although mitochondrial dysfunction can be the result of direct damage to primary oxidative phosphorylation, it is more often due to secondary involvement of mitochondrial dynamics (e.g., size, shape, distribution, movement, fission-fusion, etc.) or quality control. This has also been suggested by the evidence that many genes causing familial forms of PD, such as *PARKIN*, *PINK1*, and *DJ-1*, are critical for mitochondrial functions, whereas insoluble α-synuclein accumulating in PD, DLB, and other synucleinopathies probably affects mitochondria directly. In addition, polymorphisms or mutations in both nuclear DNA and mitochondrial DNA (mtDNA) have been implicated in the development of PD and DLB, among others, including individuals at risk for these conditions.

**Figure 1:**
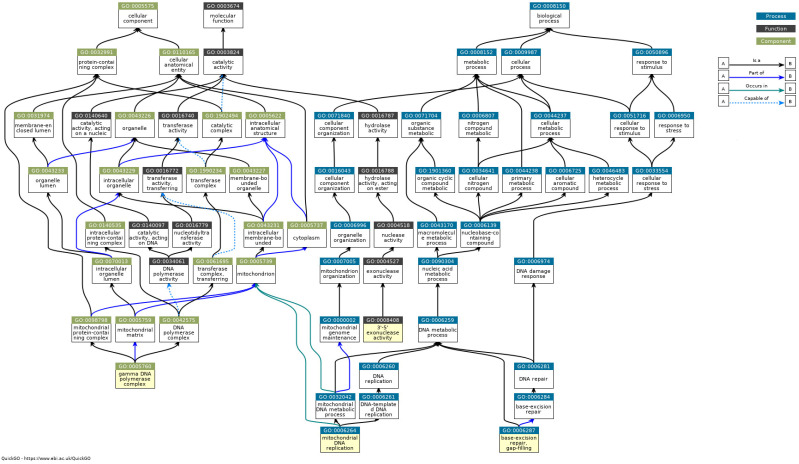
Gene ontology term assignment of POLG protein. Gene ontology terms were assigned across three categories: cellular component (GO:0005760, GO:0005739), biological process (GO:0006287, GO:0006264), and molecular function (GO:0003887, GO:0008408) from QuickGO (https://www.ebi.ac.uk/QuickGO (accessed on 12 March 2023)), which integrates diverse biological annotation sources to assign ontology terms. Of particular interest are the annotations related to restoring DNA after damage, including GO:0006281 (DNA repair) and GO:0006287 (base-excision repair, gap-filling).

In this scenario, it is worth noting that DNA polymerase γ, the only DNA polymerase located within mitochondria, is essential for mtDNA replication and repair [17]. Human DNA polymerase γ is a heterotrimeric protein complex with a catalytic subunit, encoded by the *POLG* gene at chromosomal locus 15q25, and a homodimeric accessory subunit, which is encoded by the *POLG2* gene at chromosomal locus 17q24.1 [18]. The catalytic subunit, a 1239 amino acid protein of 140 kDa, has three enzymatic activities: DNA polymerase activity, responsible for replication; 3′-5′ exonuclease activity, which proofreads misincorporated nucleotides; and 5′-deoxyribose phosphate lyase activity, required for base excision repair (Figure 1). This subunit contains an amino-terminal exonuclease domain (26–418 amino acids) connected by a linker region (419–755 amino acids) to the carboxy-terminal polymerase domain (756–1239 amino acids), with a well-conserved sequence across species. As such, *POLG* gene mutations alter the stability of mtDNA and cause several clinical phenotypes, such as dysarthria and ophthalmoplegia (SANDO), progressive external ophthalmoplegia (PEO), spinocerebellar ataxia and epilepsy (SCAE), Alpers syndrome, and sensory ataxic neuropathy. The current literature describes approximately 300 pathogenic variants in the *POLG* gene [19], with recent evidence indicating that these mutations also occur in the abovementioned neurodegenerative disorders [20], but systematic screening in these types of patients is currently lacking.

In PD, different studies have shown an elevated frequency of rare alleles of the *POLG* CAG-repeat (poly-Q), thus suggesting a possible involvement of this allelic variation in PD [21,22]. It is interesting to note that mutations in this gene have been found in patients with both familial parkinsonism and mitochondrial diseases. In 2006, Davidzon et al. [23] reported two sisters with early-onset parkinsonism and neuropathy who were compound heterozygous for a novel G737R mutation and an R853W mutation in the *POLG* gene. In 2019, Ma et al. [24] described a female compound heterozygous for a novel I898T mutation and S998L that presented with levodopa-responsive parkinsonism, external ophthalmoplegia, and optic atrophy. Finally, Mancuso et al. [25] reported a proband and her brother with the heterozygous Y831C mutation that presented with parkinsonism and PEO. However, the pathogenic role of this mutation is still unclear; indeed, although the Y831C mutation has been observed in heterozygous or compound heterozygous conditions in both infantile and late-onset cases with variable phenotypes, it has also been shown to be present at a high frequency in healthy controls [26,27].

In this study, we screened a sample of 33 patients presenting with neurodegenerative diseases, including PD, some atypical parkinsonisms, and dementia of different types, to further explore the frequency of *POLG* gene mutations in these disorders and to elucidate their role in the complex network involving neurodegeneration and mitochondrial dysfunction.

## 2. Materials and Methods

The sample included a total of 33 patients (9 females; mean age 70.1 years, standard deviation 8.2 years) with different neurodegenerative disorders. Detailed clinical, neuroradiological, neuropsychological, and genetic investigations were carried out, and the relevant demographic and clinical features are summarized in Table 1. All participants were Caucasian, of Sicilian ancestry. Their age at the time of examination was between 52 and 92 years, whereas their age at disease onset ranged from 49 to 85 years. Family history was collected through a detailed interview with a first-degree relative or the proband’s partner. The clinical and past medical histories of each patient were collected, and all the available documents related to the patients and other affected family members (e.g., medical records, certificates, and drug prescriptions) were acquired. In 11 patients, a family history of neurodegenerative disease was reported, whereas the other cases were considered sporadic, as determined by the patients’ recall and the caregivers’ confirmation.

The presence of a neurodegenerative dementia (i.e., AD, DLB, FTD) and/or movement disorder (i.e., PD, atypical parkinsonisms) was considered an inclusion criterion, whereas patients with secondary forms of dementia or movement disorders, as well as those with other neurological or neuropsychiatric diseases, were excluded. The additional exclusion criteria were: age < 18 years, cognitive impairment/disorder without dementia, and refusal to provide informed consent. All clinical diagnoses were supplied by two trained neurologists (G.L. and M.C.), in accordance with current diagnostic criteria.

In order to identify mutations in the *POLG* gene (RefSeq NM_002693.1), the genomic DNA of the patients was extracted from peripheral blood using standard protocols. PCR and sequencing reactions were performed as described by Scuderi et al. [28]. Briefly, PCR products were sequenced using a BigDye Terminator v1.1 Cycle Sequencing Kit (Thermo Fisher Scientific, Vilnius, Lithuania), purified with a DyeEx 2.0 Spin Kit (Qiagen, Hilden, Germany) that remove any type of dye terminator and analyzed on an ABI310 automated DNA sequencer. Only variants with a minor allele frequency of ≤0.01 in the Exome Aggregation Consortium (ExAC, http://exac.broadinstitute.org (accessed on 11 January 2023)), 1000 Genomes Project (http://1000genomes.org (accessed on 11 January 2023)), or Genome Aggregation Database (gnomAD, https://gnomad.broadinstitute.org/ (accessed on 11 January 2023)) were considered. A deleterious single nucleotide variant was predicted by the SIFT (http://sift.bii.a-star.edu.sg/ (accessed on 11 January 2023)), PolyPhen-2 (http://genetics.bwh.harvard.edu/pph2/ (accessed on 11 January 2023)), and Mutation Taster (http://www.mutationtaster.org/ (accessed on 11 January 2023)) programs. Mutant and normal protein structures were compared using 3D modeling. Molecular graphics and analyses were performed with UCSF Chimera 1.16 (https://www.rbvi.ucsf.edu/chimera (accessed on 23 February 2023)). To evaluate the pathogenic nature of the identified mutations, a literature review was carried out based on research in the online tools Human DNA Polymerase Gamma Mutation Database, PubMed, and Human Gene Mutation Database.

The study was conducted in accordance with the Declaration of Helsinki of 1964 and its later amendments, and the protocol was approved by the Ethics Committee of the Oasi Research Institute–IRCCS of Troina (Italy) on 5 April 2022 (approval code: 2022/04/05/CE-IRCCS-OASI/52). All subjects or their legal guardians gave informed consent for inclusion before they participated in the study.

## 3. Results

**Figure 2:**
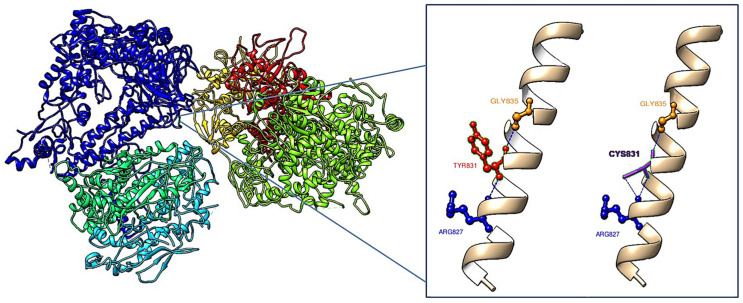
Comparison of 3D models for the normal POLG protein and POLG Y831C mutation, highlighting altered intramolecular interactions. In the normal protein, Tyr831 forms a hydrogen bond with Gly835 and Arg827. In the mutated protein, instead, Cys831 maintains a hydrogen bond with Gly835 but forms two hydrogen bonds with Arg827.

Sequence analysis of the *POLG* gene revealed the known heterozygous mutation c.2492A>G in exon 16, which causes amino acid change Tyr831Cys (Y831C), in two patients, one with FTD and one with DLB. This variant has an allele frequency of 0.00628 from the ExAC, 0.00220 from the 1000 Genomes Project, and 0.00708 from gnomAD, and was also predicted to be probably damaging by PolyPhen-2, deleterious by SIFT, and disease causing by Mutation Taster. The 3D modeling of mutant and normal protein structures (Figure 2) showed changes in intramolecular interactions that might lead to abnormal conformation. In particular, Tyr831 forms a hydrogen bond with both Gly835 and Arg827. Cys831 maintains the hydrogen bond with Gly835 but has a strong interaction with Arg827 (two hydrogen bonds).

**Figure 3:**
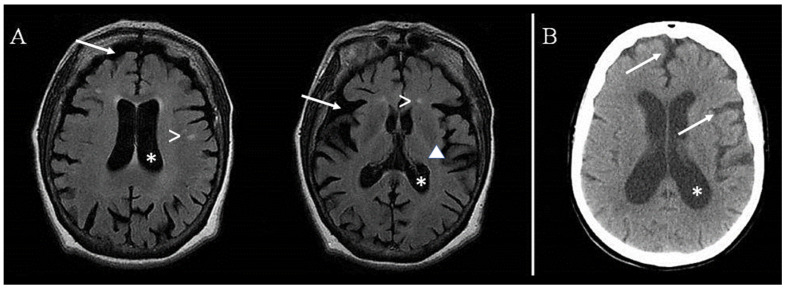
(**A**) Case 1: brain magnetic resonance imaging showing cerebral cortical atrophy, especially in the frontal lobes and perisilvian spaces (arrows), slight ventricular enlargement (asterisks), widening of the perivascular spaces (especially at the insular and nuclear-capsular levels, bilaterally) (**▲**), and mild signs of microangiopathy of the cerebral white matter (**>**). (**B**) Case 2: brain computed tomography scan showing diffuse cortical and subcortical atrophy (arrows), along with ectasia of the lateral ventricles (asterisk).

Case 1 (patient no. 1) was a 60-year-old woman with a diagnosis of FTD. The family history was positive for cognitive disorders, as the mother had been affected by cognitive decline (not better specified), the father had probably been affected by vascular cognitive impairment, and a brother had a reported diagnosis of FTD. Clinically, at the age of 54, she presented with progressive changes in personality and behavior, apathy, memory loss, cognitive decline, logorrhea, perseveration, mood changes (mainly depression), delusional ideation (mainly with religious content), and complex visual hallucinations (mostly with a mystical background). Subsequently, significant dysphagia appeared for both solids and liquids and, at the age of 59, she presented with two episodes of loss of consciousness, gaze fixation, skin pallor, and loss of muscle tone that lasted for a few minutes. At the time of our observation, she was disoriented in space and time and presented with severe cognitive decline, abnormal gait, and parkinsonism. She was treated with olanzapine, tetrabenazine, valproic acid, and lorazepam. Brain magnetic resonance imaging (Figure 3A) showed diffuse cerebral cortical atrophy, especially in the frontal lobes and perisilvian spaces, slight ventricular enlargement, widening of the perivascular spaces (especially at the insular and nuclear-capsular regions, bilaterally), and mild signs of microangiopathy of the cerebral white matter.

Case 2 (patient no. 12) was an 82-year-old woman diagnosed with DLB who was also affected by hypertension and bilateral sensorineural hearing loss. The family history was positive for cognitive decline (not further specified) and schizophrenia. At the age of 77, she started to complain about episodes of disorientation in space and time and short-term memory lapses.

In the subsequent two months, the relatives reported a rapid worsening of memory and behavioral disturbances (i.e., aggressiveness and opposite behavior), complex hallucinations, postural instability with falls, and clumsy gait; an episode of delirium also occurred. Clinically, she showed multiple severe neurocognitive deficits associated with behavioral changes and parkinsonism. A brain computed tomography scan (Figure 3B) showed diffuse cortical and subcortical atrophy, along with ectasia of the lateral ventricles.

## 4. Discussion

Our report aimed to identify POLG involvement in a sample of 33 patients presenting with neurodegenerative disorders, including PD, some atypical parkinsonisms, and dementia of different types. Mutational analysis revealed the presence of the heterozygous Y831C mutation in two patients, one with FTD and one with DLB.

The Y831C mutation was first reported by Barthelemy et al. [26] in a subject with severe mtDNA depletion. Notwithstanding the relevant change in the amino acid sequence (tyrosine to cysteine), this mutation was considered nonpathogenic for several reasons: it was located outside the functional region of the protein, it involved a poorly-conserved amino acid, and it was identified not only in the proband but also in her mother and grandmother who were both clinically healthy at the time of observation and without muscular mtDNA depletion. Moreover, the authors found the same heterozygous mutation at a frequency of 57% in 87 healthy French controls. Conversely, Mancuso et al. [25] reported a Y831C mutation in a family with late-onset PEO, parkinsonism, and peripheral neuropathy. The authors considered this mutation pathogenic since it was compatible with the clinical presentation of both PEO and peripheral neuropathy, and the mutated amino acid was very close to motif A in the polymerase region of POLG; additionally, this mutation was not found in 130 healthy controls. 

Then, Wong et al. [29] and Woodbridge et al. [30] reported, respectively, six and two heterozygous cases of Y831C mutation with variable features: hypotonia, autism, neuropathy, ataxia, hearing loss, short stature, muscle weakness, fatigue, development delay or dementia, seizures, and PEO. A case with headache, myopathy, and ischemic stroke was described by Min et al. [31], and two patients (one with myopathy and one with PEO) were reported by Da Pozzo et al. [32]. The heterozygous Y831C mutation was also found in all affected members of a South African family with arrhythmogenic cardiomyopathy (ACM) and left ventricular fibrosis [33]. Due to the ambiguous role of Y831C, the authors investigated zebra fish embryos and observed that larvae expressing the mutated *POLG* gene (c.2492A>G) developed abnormal hearts. Although the overexpression model used in that study had limitations and did not fully replicate the heterozygous disease state, these findings suggested that POLG variation may have a mechanistic role in the development of ACM. Taken together, these clinical observations demonstrate variable disease expression and age of onset associated with Y831C, as shown in Table 2.

Some population studies (Table 3), however, have questioned the pathogenic role of the Y831C mutation [34,39]. Namely, Stopińska et al. in 2006 [27] screened the Polish population for this mutation and found it in blood samples from 3 out of 133 randomly selected, clinically healthy individuals (frequency of 2.25%). It should be noted that the allele frequency of the Y831C mutation reported by the 1000 Genomes Project in the healthy population is 0.22%, whereas in the present study, the mutation was found in 2 out of 33 patients, or an allele frequency of 3.03%. More importantly, it was absent in 100 healthy controls, thus demonstrating a statistically significant difference between the two groups.

The Y831C mutation was also reported in trans with a second novel mutation (H1134R) in a case of infantile hepatocerebral mtDNA depletion syndrome; each of her asymptomatic parents carried one of the two mutations [35]. Subsequently, Hikmat et al. [37] described the same two mutations in a case of infantile myocerebrohepatopathy spectrum disorders. Notably, the Y831C mutation was observed in association with another pathogenic variant in the *POLG* gene (c.156_158dupGCA, p.Q52dup) in a patient with late-onset unclassified epileptic encephalopathy who presented with both electrical and neuroimaging posterior brain abnormalities; overall, this was compatible with those previously described in patients harboring variants in the *POLG* gene [38].

Finally, other findings come from studies on psychiatric disorders. Kasahara et al. [41] examined the impact of POLG variants in Japanese patients with bipolar disorder (BD) on protein function using three different modalities (i.e., in silico prediction, biochemical assays, and clinical evaluation) and on several other variants, including Y831C. Most of the POLG variants located in the linker domain and polymerase domain were deemed to be deleterious based on either biochemical analyses or in silico predictions. These variants lacked both exonuclease and polymerase activities, unlike variants in the exonuclease domain, which only affected exonuclease activity. In particular, for the Y831C mutation, the overall evaluation showed a mild reduction in protein activity, which was not observed in BD patients (*n* = 796) or in healthy controls (*n* = 767).

In addition, as shown by 3D modeling, the exchange from native amino acid to Cys831 changed the intramolecular interactions with Gly835 and Arg827, thus possibly leading to an abnormal protein conformation and, as a consequence, a reduction in the catalytic activity of the protein. Moreover, the presence of the Cys831 mutation may increase the risk of abnormal intramolecular disulfide bond formation with another Cys. These new S-S bonds might give rise to novel inter- or intramolecular structures, eventually contributing to a reduction in protein functionality. According to these observations, it can be assumed that the reduction in the catalytic activity of the polymerase, determined by the Y831C mutation, may result in an increase in mtDNA mutations. Impaired base-excision repair, as indicated by the GO terms “GO:0006281 DNA repair” and “GO:0006287 base-excision repair, gap-filling” assigned to POLG, may contribute to the accumulation of these mtDNA mutations. These changes would impact respiratory function, causing ATP failure and increased ROS, which would ultimately contribute to further mtDNA damage, energetic failure of neuronal cells, and subsequent degeneration.

The main limitation of this in vivo study is the lack of tissue-specific functional investigations that might have fully confirmed the hypothesis of pathogenicity for this mutation. Additionally, none of the family members could be sequenced for Y831C (unavailable/not contactable or refused to perform genetic testing). Finally, future studies with follow-up assessments are needed to further validate these results and interpretations.

## 5. Conclusions

Overall, these results may expand the genotype-phenotype spectrum associated with mutations in the *POLG* gene and strengthen the hypothesis of a pathogenetic role of the Y831C mutation in neurodegeneration presenting with different phenotypes, probably due to other genetic and/or environmental factors. Future observations are needed.

## Figures and Tables

**Table 1:** Relevant demographic and clinical features of the patients included in the study.

N.	Sex	Age Years	Age at Onset Years	Diagnosis	Family History	Neurological and/or Neuropsychiatric Comorbidities
1	F	60	54	FTD	Yes (mother)	Parkinsonism, lacunar CVD
2	M	76	67	PD	No	Mild dementia, RBD, OSAS, chronic diffuse CVD
3	M	70	60	AD	Yes (mother)	Chronic diffuse CVD
4	M	69	63	DLB	No	None
5	F	65	60	CBD	No	Mild dementia, lacunar CVD
6	M	60	58	FTD	Yes (NS)	Parkinsonism, lacunar CVD
7	M	73	69	PD	No	RBD, OSAS, lacunar CVD
8	F	83	77	AD	No	Parkinsonism, chronic diffuse CVD
9	M	67	59	DLB	No	None
10	M	68	64	PD	No	MCI, depression, lacunar CVD
11	M	68	65	PD	No	MCI, depression
12	F	82	52	DLB	Yes (NS)	Parkinsonism, chronic diffuse CVD, schizophrenia
13	M	80	69	PD	No	MCI, obsessive-compulsive disorder, chronic diffuse CVD
14	M	92	85	DLB	No	Lacunar CVD
15	M	68	62	PSP	Yes (NS)	MCI, depression
16	F	71	68	PD	No	Dementia, OSAS, chronic diffuse CVD
17	M	62	61	PD	Yes (NS)	Lacunar CVD
18	M	80	77	PD	Yes (NS)	MCI, lacunar CVD
19	M	71	78	PD	No	RBD, chronic diffuse CVD
20	M	74	70	DLB	No	Parkinsonism, RBD, OSAS
21	M	52	49	PD	No	RBD, lacunar CVD
22	M	77	72	PD	Yes (NS)	RBD, OSAS, narcolepsy, chronic diffuse CVD
23	M	69	66	FTD	No	Previous traumatic brain injury
24	M	76	72	DLB	Yes (NS)	Parkinsonism, previous stroke
25	M	76	75	PD	No	Lacunar CVD
26	F	70	66	DLB	Yes (NS)	Parkinsonism, RBD, chronic diffuse CVD
27	F	69	67	AD	No	Lacunar CVD
28	F	63	58	PD	Yes (NS)	Lacunar CVD, distal motor-sensory polyneuropathy
29	M	57	51	AD	No	Depression
30	M	66	56	PD	No	RBD, chronic diffuse CVD, anxiety disorder
31	M	60	57	PD	No	RBD, insomnia, chronic diffuse CVD,
32	M	71	63	PD	No	MCI, RBD, lacunar CVD
33	F	67	60	PD	No	MCI, previous stroke, chronic diffuse CVD

Legend (in alphabetical order): AD: Alzheimer’s disease; CBD: cortico-basal degeneration; CVD: cerebrovascular disease; DLB: dementia with Lewy bodies; F: female; FTD: frontotemporal dementia; M: male; MCI: mild cognitive impairment; NS: not specified; OSAS: obstructive sleep apnea syndrome; PD: Parkinson’s disease; PSP: progressive supranuclear palsy; RBD: REM sleep behavior disorder.

**Table 2:** Genetic characteristics and clinical phenotypes of cases reported in the literature to date.

Allele 1	Allele 2	Sex	Age at Onset (Years)	Ocular Abnormalities	Cognitive Deficit	Epilepsy	Myopathy	Movement Disorder	Peripheral Neuropathy	Hepatic Involvement	Other Signs	Reference
Y831C	-	F	6	Optic atrophy, ophthalmoplegia, retinitis pigmentosa	Cognitive impairment	+	Exercise intolerance, persistent muscle weakness, diffuse myalgia, respiratory distress	-	-	-	Severe sensorineural hearing loss, nephrotic syndrome, severe mtDNA depletion, mtDNA multiple deletions and heteroplasmic point mutations	[26]
Y831C	-	F	28	PEO	-	-	-	Parkinsonism	+	-	Hypertension and gonadal dysgenesis, mtDNA multiple deletions	[25]
Y831C Q1236H	-	F	70	-	-	NA	-	PD	-	NA	Ischemic heart disease, atrial fibrillation, hypertension	[34]
Y831C	-	F	0.1	NA	NA	NA	NA	NA	NA	NA	-	[29]
Y831C	-	M	0.1	-	DD	+	Hypotonia	-	-	-	-	[29]
Y831C	-	M	1.8	-	-	-	Hypotonia	-	-	-	Elevated lactate level, autism	[29]
Y831C	-	F	9	-	DD	+	-	-	-	-	-	[29]
Y831C	-	F	14	NA	NA	NA	NA	NA	NA	NA	-	[29]
Y831C	-	M	19	PEO	DD	-	Muscle weakness, fatigue	Ataxia	+	-	Hearing loss, short stature	[29]
Y831C	H1134R	F	0.3	NA	NA	NA	NA	NA	NA	Mildly icteric with hepatomegaly	Anorexia, failure to thrive syndrome, drowsiness, peripheral edema, mtDNA depletion	[35]
Y831C	R722H	F	56	-	NA	NA	NA	PD	NA	NA	Hyperreflexia	[36]
Y831C	-	F	34	C-PEO	NA	+	+	Ataxia	+	+	Dysphagia/dysarthria, gastric and small bowel dysfunction, sensorineural hearing loss, palatal insufficiency, mtDNA multiple deletions	[30]
Y831C	-	F	46	Ptosis	NA	+	Myokymia, muscle weakness, myalgia	-	-	+	Migraine	[30]
Y831C	-	F	59	Visual deficit	Mild cognitive impairment	-	Fatigue	-	-	-	Ischemic stroke in the left occipital and parietal lobes, hypertension, hyperlipidaemia, intermittent limb paraesthesia, migraine-like headache, episodic confusion, dysarthria	[31]
Y831C	-	M	72	-	-	-	Axial myopathy	-	-	-	-	[32]
Y831C	-	F	59	Ptosis	-	-	-	-	-	-	Thyreopathy, hearing loss	[32]
Y831C	H1134R	NA	0.1	Visual deficit	DD	-	Hypotonia	-	-	+	Failure to thrive syndrome, altered growth, renal dysfunction, MCHS	[37]
Y831C	Q52dup	M	3	NA	DD	+	NA	NA	NA	NA	Epileptic encephalopathy with posterior electrical and neuroimaging abnormalities, SRPX2 mutation	[38]
Y831C	-	M	50	NA	NA	NA	NA	NA	NA	NA	Arrhythmogenic cardiomyopathy and left ventricular fibrosis	[33]

Legend (in alphabetical order): C-PEO: chronic-progressive external ophthalmoplegia; CPK: creatinine phosphokinase; DD: developmental delay; F: female; M: male; MCHS: myocerebrohepatopathy spectrum disorders; mtDNA: mitochondrial DNA; NA: not available; PD: Parkinson’s disease; +: present; -: absent.

**Table 3:** Frequencies of the Y831C mutation in population-based case-control studies.

Study Population	Frequency		Reference
	Patients	Controls	
Healthy Polish population	-	2.25% (3/133)	[27]
British and Italian idiopathic patients with Parkinson’s disease	2.14% (3/140 British)	3.33% (3/90 British) *	[39]
	0.00% (0/279 Italian)	0.00% (0/285 Italian)	
Finnish patients with Parkinson’s disease	0.71% (1/140)	3.94% (5/127) *	[34]
Chinese sporadic patients with Parkinson’s disease	8.14% (28/344)	11.04% (17/154) *	[40]
Japanese patients with bipolar disorder	0.00% (0/796)	0.00% (0/767)	[41]
Present study	6.06% (2/33)	0.00% (0/100)	-

* age- and ethnically-matched.

## Data Availability

No new data were created or analyzed in this study. Data sharing is not applicable to this article.

## References

[1] Zhou J., Gennatas E.D., Kramer J.H., Miller B.L., Seeley W.W. (2012). Predicting Regional Neurodegeneration from the Healthy Brain Functional Connectome. Neuron.

[2] Salemi M., Lanza G., Mogavero M.P., Cosentino F.I.I., Borgione E., Iorio R., Ventola G.M., Marchese G., Salluzzo M.G., Ravo M. (2022). A Transcriptome Analysis of MRNAs and Long Non-Coding RNAs in Patients with Parkinson’s Disease. Int. J. Mol. Sci..

[3] Abeliovich A., Gitler A.D. (2016). Defects in Trafficking Bridge Parkinson’s Disease Pathology and Genetics. Nature.

[4] Canter R.G., Penney J., Tsai L.-H. (2016). The Road to Restoring Neural Circuits for the Treatment of Alzheimer’s Disease. Nature.

[5] Taylor J.P., Brown R.H., Cleveland D.W. (2016). Decoding ALS: From Genes to Mechanism. Nature.

[6] Wyss-Coray T. (2016). Ageing, Neurodegeneration and Brain Rejuvenation. Nature.

[7] Lanza G., Centonze S.S., Destro G., Vella V., Bellomo M., Pennisi M., Bella R., Ciavardelli D. (2018). Shiatsu as an Adjuvant Therapy for Depression in Patients with Alzheimer’s Disease: A Pilot Study. Complement. Ther. Med..

[8] Cantone M., Lanza G., Ranieri F., Opie G.M., Terranova C. (2021). Editorial: Non-Invasive Brain Stimulation in the Study and Modulation of Metaplasticity in Neurological Disorders. Front. Neurol..

[9] Lanza G., Casabona J.A., Bellomo M., Cantone M., Fisicaro F., Bella R., Pennisi G., Bramanti P., Pennisi M., Bramanti A. (2020). Update on Intensive Motor Training in Spinocerebellar Ataxia: Time to Move a Step Forward?. J. Int. Med. Res..

[10] Caruso G., Godos J., Privitera A., Lanza G., Castellano S., Chillemi A., Bruni O., Ferri R., Caraci F., Grosso G. (2022). Phenolic Acids and Prevention of Cognitive Decline: Polyphenols with a Neuroprotective Role in Cognitive Disorders and Alzheimer’s Disease. Nutrients.

[11] Ilieva H., Polymenidou M., Cleveland D.W. (2009). Non-Cell Autonomous Toxicity in Neurodegenerative Disorders: ALS and Beyond. J. Cell Biol..

[12] Marambaud P., Dreses-Werringloer U., Vingtdeux V. (2009). Calcium Signaling in Neurodegeneration. Mol. Neurodegener..

[13] Kiaei M. (2013). New Hopes and Challenges for Treatment of Neurodegenerative Disorders: Great Opportunities for Young Neuroscientists. Basic Clin. Neurosci..

[14] Johri A., Beal M.F. (2012). Mitochondrial Dysfunction in Neurodegenerative Diseases. J. Pharmacol. Exp. Ther..

[15] Briston T., Hicks A.R. (2018). Mitochondrial Dysfunction and Neurodegenerative Proteinopathies: Mechanisms and Prospects for Therapeutic Intervention. Biochem. Soc. Trans..

[16] Muddapu V.R., Dharshini S.A.P., Chakravarthy V.S., Gromiha M.M. (2020). Neurodegenerative Diseases—Is Metabolic Deficiency the Root Cause?. Front. Neurosci..

[17] Lanza G., Cantone M., Musso S., Borgione E., Scuderi C., Ferri R. (2018). Early-Onset Subcortical Ischemic Vascular Dementia in an Adult with MtDNA Mutation 3316G > A. J. Neurol..

[18] Yakubovskaya E., Chen Z., Carrodeguas J.A., Kisker C., Bogenhagen D.F. (2006). Functional Human Mitochondrial DNA Polymerase Gamma Forms a Heterotrimer. J. Biol. Chem..

[19] Nicholas Russo S., Shah E.G., Copeland W.C., Koenig M.K. (2022). A New Pathogenic POLG Variant. Mol. Genet. Metab. Rep..

[20] Hsieh P.-C., Wang C.-C., Tsai C.-L., Yeh Y.-M., Lee Y.S., Wu Y.-R. (2019). POLG R964C and GBA L444P Mutations in Familial Parkinson’s Disease: Case Report and Literature Review. Brain Behav..

[21] Eerola J., Luoma P.T., Peuralinna T., Scholz S., Paisan-Ruiz C., Suomalainen A., Singleton A.B., Tienari P.J. (2010). POLG1 Polyglutamine Tract Variants Associated with Parkinson’s Disease. Neurosci. Lett..

[22] Anvret A., Westerlund M., Sydow O., Willows T., Lind C., Galter D., Belin A.C. (2010). Variations of the CAG Trinucleotide Repeat in DNA Polymerase γ (POLG1) Is Associated with Parkinson’s Disease in Sweden. Neurosci. Lett..

[23] Davidzon G., Greene P., Mancuso M., Klos K.J., Ahlskog J.E., Hirano M., DiMauro S. (2006). Early-Onset Familial Parkinsonism Due to POLG Mutations. Ann. Neurol..

[24] Ma L., Mao W., Xu E., Cai Y., Wang C., Chhetri J.K., Chan P. (2020). Novel POLG Mutation in a Patient with Early-Onset Parkinsonism, Progressive External Ophthalmoplegia and Optic Atrophy. Int. J. Neurosci..

[25] Mancuso M., Filosto M., Bellan M., Liguori R., Montagna P., Baruzzi A., DiMauro S., Carelli V. (2004). POLG Mutations Causing Ophthalmoplegia, Sensorimotor Polyneuropathy, Ataxia, and Deafness. Neurology.

[26] Barthélémy C., de Baulny H.O., Lombès A. (2002). D-Loop Mutations in Mitochondrial DNA: Link with Mitochondrial DNA Depletion?. Hum. Genet..

[27] Stopińska K., Grzybowski T., Malyarchuk B.A., Derenko M.V., Miścicka-Sliwka D. (2006). Optimization of the Y831C Mutation Detection in Human DNA Polymerase Gamma by Allelic Discrimination Assay. Acta Biochim. Pol..

[28] Scuderi C., Borgione E., Castello F., Lo Giudice M., Santa Paola S., Giambirtone M., Di Blasi F.D., Elia M., Amato C., Città S. (2015). The in Cis T251I and P587L POLG1 Base Changes: Description of a New Family and Literature Review. Neuromuscul. Disord..

[29] Wong L.-J.C., Naviaux R.K., Brunetti-Pierri N., Zhang Q., Schmitt E.S., Truong C., Milone M., Cohen B.H., Wical B., Ganesh J. (2008). Molecular and Clinical Genetics of Mitochondrial Diseases Due to POLG Mutations. Hum. Mutat..

[30] Woodbridge P., Liang C., Davis R.L., Vandebona H., Sue C.M. (2013). POLG Mutations in Australian Patients with Mitochondrial Disease. Intern. Med. J..

[31] Min J., Farooq M.U., Glisson C. (2014). Adult Phenotypic Spectrum of Headache, Myopathy and Ischemic Stroke Associated with Mitochondrial POLG Mutation. Austin J. Cerebrovasc. Dis. Stroke.

[32] Da Pozzo P., Cardaioli E., Rubegni A., Gallus G.N., Malandrini A., Rufa A., Battisti C., Carluccio M.A., Rocchi R., Giannini F. (2017). Novel POLG Mutations and Variable Clinical Phenotypes in 13 Italian Patients. Neurol. Sci..

[33] Spracklen T.F., Kasher P.R., Kraus S., Botha T.L., Page D.J., Kamuli S., Booi Z., Chin A., Laing N., Keavney B.D. (2021). Identification of a POLG Variant in a Family with Arrhythmogenic Cardiomyopathy and Left Ventricular Fibrosis. Circ. Genom. Precis. Med..

[34] Luoma P.T., Eerola J., Ahola S., Hakonen A.H., Hellström O., Kivistö K.T., Tienari P.J., Suomalainen A. (2007). Mitochondrial DNA Polymerase Gamma Variants in Idiopathic Sporadic Parkinson Disease. Neurology.

[35] Taanman J.-W., Rahman S., Pagnamenta A.T., Morris A.A.M., Bitner-Glindzicz M., Wolf N.I., Leonard J.V., Clayton P.T., Schapira A.H.V. (2009). Analysis of Mutant DNA Polymerase Gamma in Patients with Mitochondrial DNA Depletion. Hum. Mutat..

[36] Ylönen S., Ylikotila P., Siitonen A., Finnilä S., Autere J., Majamaa K. (2013). Variations of Mitochondrial DNA Polymerase γ in Patients with Parkinson’s Disease. J. Neurol..

[37] Hikmat O., Tzoulis C., Chong W.K., Chentouf L., Klingenberg C., Fratter C., Carr L.J., Prabhakar P., Kumaraguru N., Gissen P. (2017). The Clinical Spectrum and Natural History of Early-Onset Diseases Due to DNA Polymerase Gamma Mutations. Genet. Med..

[38] Ortega-Moreno L., Giráldez B.G., Soto-Insuga V., Losada-Del Pozo R., Rodrigo-Moreno M., Alarcón-Morcillo C., Sánchez-Martín G., Díaz-Gómez E., Guerrero-López R., Serratosa J.M. (2017). Molecular Diagnosis of Patients with Epilepsy and Developmental Delay Using a Customized Panel of Epilepsy Genes. PLoS ONE.

[39] Tiangyou W., Hudson G., Ghezzi D., Ferrari G., Zeviani M., Burn D.J., Chinnery P.F. (2006). POLG1 in Idiopathic Parkinson Disease. Neurology.

[40] Gui Y., Xu Z., Lv W., Liu H., Zhao J.-J., Hu X.-Y. (2012). Association of Mitochondrial DNA Polymerase γ Gene POLG1 Polymorphisms with Parkinsonism in Chinese Populations. PLoS ONE.

[41] Kasahara T., Ishiwata M., Kakiuchi C., Fuke S., Iwata N., Ozaki N., Kunugi H., Minabe Y., Nakamura K., Iwata Y. (2017). Enrichment of Deleterious Variants of Mitochondrial DNA Polymerase Gene(POLG1) in Bipolar Disorder. Psychiatry Clin. Neurosci..

